# Effectiveness of a Nurse-Led Brief Psychosocial Intervention on Harmful Alcohol Use Among Indian Undergraduate Students

**DOI:** 10.7759/cureus.95384

**Published:** 2025-10-25

**Authors:** Beoma Pandey, Pity Koul

**Affiliations:** 1 Nursing, Sharda School of Nursing Science and Research, Sharda University, Greater Noida, IND

**Keywords:** brief intervention, frames model, harmful alcohol use, nurse-led intervention, undergraduate students

## Abstract

Background: Harmful alcohol use among undergraduate students is a rising concern, with implications for physical, psychological, and academic outcomes. Evidence for nurse-led brief psychosocial interventions based on the FRAMES (Feedback, Responsibility, Advice, Menu of options, Empathy, and Self-efficacy) model remains limited in Indian settings.

Methods: A quasi-experimental study was conducted among 330 undergraduate students identified as harmful alcohol users using the Alcohol Use Disorders Identification Test (AUDIT), allocated into experimental (n = 162) and control (n = 168) groups. Baseline data were collected using the AUDIT tool and sociodemographic questionnaire. The experimental group received two sessions of one-on-one brief negotiated interviews using FRAMES, delivered by a trained nursing professional. Post-test data were collected after three and six months. Non-parametric tests were used for within- and between-group comparisons.

Results: At baseline, sociodemographic characteristics and mean AUDIT scores were comparable between the experimental and control groups. Following the intervention, the experimental group showed a significant reduction in mean AUDIT scores (p < 0.001), while the control group did not show any notable changes.

Conclusion: Nurse-led FRAMES-based brief psychosocial intervention effectively reduces harmful alcohol use among undergraduate students. Given its brevity, feasibility, and potential scalability, this model may be incorporated into college-based health services for early identification and intervention.

## Introduction

Alcohol use among college and university students in India is becoming an increasing threat to public health [1]. Given that a sizable portion of India's population is young, trends of students starting to drink alcohol too young and engaging in risky drinking are becoming increasingly evident [1,2]. Recent regional studies from Kerala and North India have raised some serious concerns, revealing that the prevalence rates of alcohol consumption among college students can range anywhere from 10% to over 30% [3-6]. This is particularly troubling when the risks involved are considered, such as poor academic performance, engaging in risky behaviors, injuries, and the potential for long-term dependence [7]. Harmful alcohol use refers to drinking that causes negative health or social consequences, while alcohol dependence is a chronic, relapsing brain disorder characterized by loss of control over alcohol use, tolerance, withdrawal symptoms, and continued use despite significant social, occupational, or health-related harms [8]. The increasing acceptance of alcohol use in youth culture, combined with limited access to counseling services and a lack of structured preventive programs, underscores the urgent need for effective, evidence-based interventions in Indian educational institutions.

While there are various global intervention models out there, brief interventions (BIs) have proven to be both effective and practical for tackling risky alcohol use among young adults [8,9]. The World Health Organization has repeatedly endorsed brief, cost-effective interventions as a viable option in low-resource environments, including places like India [10]. Typically, BIs involve personalized feedback, motivational interviewing, and goal-setting, all delivered in short sessions. These interventions tend to work best when offered in convenient settings, such as college health clinics [11].

One approach that is really gaining attention is the integration of nurse-led BIs in university and college environments. Nurses are not only accessible and trusted, but they also have the training in health communication that makes them perfect for delivering these kinds of interventions. A randomized clinical trial conducted in India shows that nurse-delivered screening and BIs can be both feasible and effective for hazardous drinkers in college settings [12]. This method not only proves to be cost-effective but also helps in early detection and timely counseling.

However, despite the clear benefits of these strategies, there have not been many large-scale studies that assess their effectiveness specifically in Indian colleges [1,2]. Most research in India tends to focus on prevalence or employs qualitative methods, which leaves a significant gap in understanding how to implement these interventions effectively [2,3]. Additionally, there are no standardized protocols for nurse-led alcohol interventions that are specifically designed for Indian youth. This study aims to fill that critical gap by evaluating the effectiveness of a structured, nurse-led brief psychosocial intervention on harmful alcohol use among undergraduate students.

## Materials and methods

Study design

This research adopted a quantitative study approach using a quasi-experimental pre- and post-test control group design. The objective was to evaluate the effectiveness of a nurse-led brief psychosocial intervention on harmful alcohol use among undergraduate students.

Study setting

The study was conducted from July 2024 to May 2025, across four colleges affiliated with a public university in Delhi, India.

Population and sampling

Undergraduate students aged ≥18 years from selected colleges were screened for harmful alcohol use. A multistage sampling design was used. Two university zones were randomly selected, and two colleges per zone were chosen by convenience sampling. Within each zone, one college was randomly assigned to the experimental group and one to the control group.

The number of students to be screened from each academic year was proportionate to the total enrollment for that year in each college. Students were approached during scheduled classes across different courses, and all present were invited to participate. After obtaining informed consent, the Alcohol Use Disorders Identification Test (AUDIT) was administered.

Inclusion criteria were students aged ≥18 years who scored 8-19 on the AUDIT, indicating harmful alcohol use, and who provided consent to participate. Exclusion criteria included students with diagnosed psychiatric illnesses and those currently receiving treatment for alcohol use disorders.

A total of 2,360 students (837 first-year, 821 second-year, and 737 third-year students) were screened, and 360 met the inclusion criteria and were enrolled in the study.

Sample size estimation

The sample size was calculated using G*Power version 3.1.9.2 (Heinrich Heine University Düsseldorf, Düsseldorf, Germany) for a two-tailed Mann-Whitney U test, with an effect size of 0.3 (small to medium), alpha level of 0.05, and power of 0.80. The minimum required sample size was 278 participants (139 per group). Based on prior research in undergraduate student populations, the study aimed to recruit 360 participants to account for anticipated attrition and ensure adequate representation across academic years and disciplines. Considering an expected prevalence of harmful alcohol use of approximately 15% among undergraduates [12], a total of 2,360 students were screened to identify 360 eligible participants (181 in each group). During the course of the study, 30 participants were lost to follow-up, resulting in a final analyzed sample of 330 participants (162 in the experimental group and 168 in the control group).

Intervention

The intervention for the experimental group involved a brief, nurse-led psychosocial approach intended to help undergraduate students reduce or abstain from harmful alcohol use. It comprised two sessions of brief negotiated interviews (BNI), based on the FRAMES (Feedback, Responsibility, Advice, Menu of options, Empathy, and Self-efficacy) model of motivational interviewing, each lasting 20-25 minutes. Individualization was achieved by tailoring the discussion to each student’s AUDIT score, level of readiness to change, personal drinking patterns, and unique psychosocial context, including academic pressures, social influences, and previous attempts to reduce alcohol use. The first BNI session provided personalized feedback based on the student’s AUDIT results, emphasized the student’s personal responsibility for behavior change, and offered a menu of options suited to their situation. The student’s confidence and ability to implement changes were reinforced to promote self-efficacy. The second session, conducted two weeks later, focused on reassessing goals, addressing individual challenges encountered, and strengthening the student’s confidence in their ability to change. The intervention was designed to be non-judgmental, brief, and easily implementable within college environments.

Data collection tools

Data on students' alcohol use were collected using the self-report version of the AUDIT developed by the WHO and a structured socio-demographic questionnaire. The AUDIT tool is validated globally for population-based alcohol screening [13]. It has a high internal reliability (Cronbach's alpha = 0.92) for use among the Indian population [14]. The AUDIT consists of 10 questions covering alcohol consumption, dependence symptoms, and alcohol-related problems. Each question is scored from 0 to 4, with total scores ranging from 0 to 40. Based on the WHO guidelines, scores of 0-7 indicate low-risk drinking, 8-19 indicate harmful or hazardous alcohol use, and 20 or above suggest possible alcohol dependence. Students with AUDIT scores of 8-19, i.e., harmful alcohol use, were included in the analysis. The sociodemographic questionnaire and the content of the brief psychosocial intervention were validated by a panel of nine experts, including psychiatrists, clinical psychologists, psychiatric social work professionals, and psychiatric nursing faculty. The experts reviewed the tools for relevance, clarity, organization, and feasibility. Experts recommended refinements in wording, order of items, and cultural suitability, which were incorporated before final use.

Data collection procedure

Data collection was conducted after obtaining approval from the Institutional Ethical Committee (Reference no. SU/SMS&R/76-A/2023/178; dated 4th September 2023) and from the administrative heads of the participating colleges. The nurse-researcher received training in the brief psychosocial intervention based on the FRAMES model through an online certification program by the Addiction Technology Transfer Center (ATTC), U.S. Department of Health and Human Services. This program is internationally recognized and accepted for professional and research purposes in India. A rolling recruitment approach was adopted for screening and intervention. Undergraduate students from the experimental group colleges were screened using the AUDIT tool in classroom settings after obtaining informed consent. Participants with AUDIT scores between 8 and 19 completed a demographic questionnaire and received two individualized brief negotiated interview sessions, each lasting 20-25 minutes, based on the FRAMES model. The sessions were conducted 15 days apart. Students in the control group followed the same screening and data collection procedures but received no intervention. Post-test assessments using the AUDIT tool were administered at three and six months to evaluate the outcomes.

Data analysis

Microsoft Excel (Microsoft Corporation, Redmond, WA) was used to enter the data, and SPSS version 25 (IBM Corp., Armonk, NY) was used for analysis. Sociodemographic variables and alcohol-related factors were summarized using descriptive statistics, including frequencies and percentages. The distribution of AUDIT scores was examined using the Shapiro-Wilk test, which indicated significant deviation from normality (p < 0.05), confirming skewness in the data. Hence, non-parametric tests were employed. The Friedman test was performed to explore the changes in AUDIT scores within the experimental and control groups across pre-test, post-test 1, and post-test 2. The Mann-Whitney U test was used to compare the experimental and control groups in terms of AUDIT at pre-test, post-test 1, and post-test 2. All statistical analyses were performed with a significance level set at p < 0.05.

## Results

The study assessed the characteristics of undergraduate students identified as harmful alcohol users. Table 1 presents the sociodemographic profile of participants in the experimental and control groups. No significant differences were observed between the groups, indicating baseline comparability in sociodemographic variables.

**Table 1:** Sociodemographic profile of undergraduate students (N = 330). PG: paying guest accommodation; INR: Indian rupees; df: degrees of freedom; ^1^: chi-squared test.

Parameters	Group		Test value (df)	p-value	Effect size
	Control (n = 168)	Experimental (n = 162)			
	Frequency (percentage)	Frequency (percentage)			
Age					
18 years	14 (8.3%)	18 (11.1%)			
19 years	21 (12.5%)	25 (15.4%)			
20 years	53 (31.5%)	41 (25.3%)	2.297 (4)	0.681^1^	0.08
21 years	44 (26.2%)	43 (26.5%)			
>21 years	36 (21.4%)	35 (21.6%)			
Gender					
Male	144 (85.7%)	131 (80.9%)	1.397 (1)	0.237^1^	0.07
Female	24 (14.3%)	31 (19.1%)			
Academic year					
1^st^ year	48 (28.6%)	38 (23.5%)			
2^nd^ year	50 (29.8%)	42 (25.9%)	2.697 (2)	0.259^1^	0.08
3^rd^ year	70 (41.6%)	82 (50.6%)			
Current residence					
Off-campus apartment/PG	77 (45.8%)	62 (38.3%)			
Shared house/flat	43 (25.6%)	57 (35.2%)	3.746 (2)	0.154^1^	0.11
Living with parents or a guardian	48 (28.6%)	43 (26.5%)			
Family structure					
Nuclear family	130 (77.4%)	131 (80.9%)	0.605 (1)	0.437^1^	0.04
Joint family	38 (22.6%)	31 (19.1%)			
Previous year's academic performance					
>71%	15 (8.9%)	12 (7.4%)			
61-70%	44 (26.2%)	36 (22.2%)	1.297 (3)	0.730^1^	0.06
51-60%	81 (48.2%)	82 (50.6%)			
<50%	28 (16.7%)	32 (19.8%)			
Monthly income of parents (INR)					
<30,000	21 (12.5%)	13 (8.0%)			
30,001-60,000	30 (17.9%)	26 (16.0%)			
60,001-1,00,000	78 (46.4%)	89 (54.9%)	5.521 (4)	0.238^1^	0.13
1,00,001-2,00,000	17 (10.1%)	21 (13.0%)			
>2,00,001	22 (13.1%)	13 (8.0%)			

In Table 2, alcohol-related behaviors of participants are outlined for both experimental and control groups. Statistical analysis revealed no significant group differences, confirming that baseline AUDIT scores and other alcohol-related behaviors were comparable across groups.

**Table 2:** Alcohol use and related factors among undergraduate students (N = 330). ^1^: Chi-squared test; ^2^: Fisher's exact test; ^3^: Mann-Whitney U test; df: degrees of freedom; PG: paying guest accommodation; AUDIT: Alcohol Use Disorders Identification Test.

Parameters	Group		Test value (df)	p-value	Effect size
	Control (n = 168)	Experimental (n = 162)			
	Frequency (percentage)	Frequency (percentage)			
Do parents take alcohol?					
Yes	105 (62.5%)	117 (72.2%)			
No	63 (37.5%)	45 (27.8%)	3.541 (1)	0.060^1^	0.10
Do friends take alcohol?					
Yes	150 (89.3%)	140 (86.4%)	0.636 (1)	0.425^1^	0.04
No	18 (10.7%)	22 (13.6%)			
Who introduced you to alcohol?					
Parent	3 (1.8%)	6 (3.7%)			
Sibling	4 (2.4%)	7 (4.3%)			
Friend from school	40 (23.8%)	45 (27.8%)			
Friend from college	83 (49.4%)	70 (43.2%)	4.581 (5)	0.469^1^	0.12
Friend from the neighborhood	22 (13.1%)	24 (14.8%)			
By own	16 (9.5%)	10 (6.2%)			
Reason for Initiation					
Peer pressure	86 (51.2%)	85 (52.5%)			
Stress	27 (16.1%)	20 (12.3%)			
Influence of media	10 (6.0%)	14 (8.6%)			
Familial influence	7 (4.2%)	3 (1.9%)	4.578 (5)	0.470^1^	0.12
For fun	25 (14.9%)	31 (19.1%)			
Boredom	13 (7.7%)	9 (5.6%)			
First drink age					
10 to 12 years	9 (5.4%)	8 (4.9%)			
13 to 14 years	36 (21.4%)	40 (24.7%)			
15 to 17 years	78 (46.4%)	82 (50.6%)	2.456 (3)	0.483^1^	0.09
>18 years	45 (26.8%)	32 (19.8%)			
Preferred beverage					
Beer	118 (70.2%)	103 (63.6%)			
Wine	13 (7.7%)	14 (8.6%)	1.727 (2)	0.422^1^	0.07
Spirits (whiskey/vodka/rum/brandy)	37 (22.0%)	45 (27.8%)			
Where do you obtain alcohol from?					
Purchase myself (with or without a fake ID)	122 (72.6%)	109 (67.3%)			
Given by friends/family	38 (22.6%)	42 (25.9%)	1.297 (2)	0.523^1^	0.06
Parties or social events	8 (4.8%)	11 (6.8%)			
Place of consumption					
Social gatherings/parties	35 (20.8%)	37 (22.8%)			
Bars/clubs/restaurants	16 (9.5%)	13 (8.0%)			
At a friend’s place/hostel/PG	117 (69.6%)	110 (67.9%)	2.473 (3)	0.583^2^	0.09
At home	0 (0.0%)	2 (1.2%)			
Mean AUDIT score (baseline)	9.49 ± 1.67	9.62 ± 1.88		0.946^3^	0.04

Table 3 shows that in the experimental group, there was a statistically significant difference across pre-test, post-test 1, and post-test 2 (p < 0.001). In contrast, the control group showed no statistically significant difference in pre- and post-test AUDIT scores (p = 0.483). These results support the effectiveness of brief psychosocial intervention in reducing alcohol usage among undergraduate students in the experimental group.

**Table 3:** Comparison of pre-test and post-test AUDIT scores among undergraduate students within the experimental and control groups (N = 330). AUDIT: Alcohol Use Disorders Identification Test; SD: standard deviation; IQR: interquartile range; S: significant at p < 0.05; NS: not significant at p < 0.05.

Group		Pre-test	Post-test 1	Post-test 2	Friedman test value	p-value
Experimental group (n = 162)	Mean ± SD	9.62 ± 1.88	5.21 ± 1.68	5.26 ± 1.95	258.000	<0.001 (S)
	Median (IQR)	9 (8-11)	5 (4-6)	5 (4-7)		
Control group (n = 168)	Mean ± SD	9.49 ± 1.67	9.45 ± 2.30	9.46 ± 2.43	1.455	0.483 (NS)
	Median (IQR)	9 (8-10)	9 (7-10)	9 (7-11)		

Data presented in Table 4 indicate that there was a significant reduction in AUDIT scores from pre-test to post-test 1 (mean change = -4.41, SD = 2.16) and from pre-test to post-test 2 (mean change = -4.36, SD = 2.38) in the experimental group, with both changes being statistically significant (p < 0.001). This indicates a sustained improvement in alcohol use behavior following the intervention.

**Table 4:** Post hoc pairwise comparisons of pre-test and post-test AUDIT scores in the experimental group (n = 162). AUDIT: Alcohol Use Disorders Identification Test; SD: standard deviation; S: significant at p < 0.05.

Timepoint comparison	Change in AUDIT score from pre-test to post-test 1 & post-test 2 in the experimental group	
	Mean (SD) of absolute change	p-value of change within the experimental group
Pre-test to post-test 1 (baseline to 3 months)	-4.41 (2.16)	<0.001 (S)
Pre-test to post-test 2 (baseline to 6 months)	-4.36 (2.38)	<0.001 (S)

Table 5 demonstrates that at post-test 1 and post-test 2, there was a statistically significant difference in AUDIT scores between the experimental group and the control group (p < 0.001). This indicates that the brief psychosocial intervention effectively reduced the alcohol usage among undergraduates in the experimental group as compared to those in the control group.

**Table 5:** Comparison of pre-test and post-test AUDIT scores among undergraduate students between the experimental and control groups (N = 330). AUDIT: Alcohol Use Disorders Identification Test; SD: standard deviation; IQR: interquartile range; S: significant at p < 0.05, NS: not significant at p < 0.05.

	Experimental group		Control group		Mann-Whitney U test value	p-value
	Mean ± SD	Median (IQR)	Mean ± SD	Median (IQR)		
Pre-test	9.62 ± 1.88	9 (8-11)	9.49 ± 1.67	9 (8-10)	13665.000	0.946 (NS)
Post-test 1	5.21 ± 1.68	5 (4-6)	9.45 ± 2.30	9 (7-10)	25251.500	<0.001 (S)
Post-test 2	5.26 ± 1.95	5 (4-7)	9.46 ± 2.43	9 (7-11)	24201.000	<0.001 (S)

## Discussion

The present study evaluated the effectiveness of a nurse-led brief psychosocial intervention in reducing harmful alcohol use among undergraduate students. The demographic data showed that the majority of participants were 21 years or older, which fits with evidence suggesting that alcohol use tends to rise during early adulthood, especially under social and academic pressures [15].

A significant number of students lived in shared hostels or off-campus housing, pointing to a possible connection between limited parental supervision and increased alcohol consumption, as highlighted in earlier research [16]. Moreover, over three-quarters of the participants came from nuclear families. While this aligns with studies that consider family structure as a potential factor influencing substance use, the relationship is often complex and not always significant [17].

Most participants had poor academic performance, with scores falling below 61% in the previous year. This supports earlier findings that link harmful alcohol use to cognitive impairments and academic decline [18,19]. Additionally, most participants came from middle-income families, which is consistent with previous research indicating that alcohol use is not limited to high-income groups and can be influenced by factors like affordability and accessibility [20].

Peer influence has turned out to be a major player in the initiation of alcohol use. A striking 89% of participants mentioned that their friends also drank alcohol, with many first trying it out through their peers instead of family. Peer pressure topped the list as the main reason for starting to drink, followed closely by stress and the desire for fun, which backs up previous research on how social norms and emotional challenges drive young people to consume alcohol [21]. Additionally, parental alcohol use was common, echoing findings that highlight the intergenerational patterns of substance use [17].

Most students reported that they began drinking between the ages of 18 and 21 years, a time often characterized by newfound independence and a spirit of experimentation [4]. Beer was the drink of choice, followed by spirits, which aligns with global trends seen in university populations [22,23]. Alarmingly, a significant number of students managed to get alcohol using fake IDs or without any age checks, raising concerns about the enforcement of legal drinking age regulations [24].

The post-test data revealed a notable and sustained drop in AUDIT scores within the experimental group, showcasing the success of the nurse-led brief psychosocial intervention based on the FRAMES model. These results are consistent with earlier studies that highlight the effectiveness of brief motivational interventions in curbing harmful drinking behaviors among young adults [12,25]. Systematic reviews have also pointed out the crucial role nurses play in delivering effective interventions to reduce harmful alcohol use, especially in settings with limited resources [26,27].

In summary, the findings suggest that brief, structured interventions, when woven into college health programs and carried out by trained nursing professionals, can be a practical and scalable way to tackle alcohol use among students. These results support continued use and evaluation of nurse-led interventions, especially in low-resource settings like India, where access to formal psychological services may be limited. Going forward, integrating nursing professionals into college health programs could be facilitated through appropriate policy-level changes, including formal appointments and training frameworks for nurses to deliver such interventions. This approach has the potential to positively impact the health of future generations by reducing risky behaviors, alcohol-related injuries, and associated hospital visits, while improving productivity and decreasing absenteeism. Economically, this translates to lower healthcare costs and improved overall societal well-being. Policy support is essential to institutionalize these roles and ensure sustainable, long-term implementation of nurse-led interventions for harmful alcohol use within educational settings.

Strengths of the study

This study addresses a timely and significant public health issue by evaluating a structured, evidence-based nurse-led intervention for harmful alcohol use among undergraduate students in India. The use of a quasi-experimental pre-test and multiple post-test assessments allowed for the measurement of both immediate and sustained effects of the intervention. The intervention was based on the FRAMES model and individualized to each participant, with validation by experts and the use of a globally recognized screening tool (AUDIT) to ensure reliability and accuracy. Inclusion of participants from multiple academic years enhanced the representativeness and generalizability of the findings. Furthermore, the study demonstrates the feasibility and practical applicability of implementing brief, structured interventions in low-resource college settings, offering a scalable model for addressing alcohol-related harms among young adults.

Limitations of the study

The study was based on self-reported measures of alcohol consumption, which may be subject to social desirability effects. The follow-up period was limited to six months post-intervention in the study. While the results indicate short-term effectiveness, the long-term sustainability of behavior change remains uncertain. Additionally, convenience sampling of colleges may limit the generalizability of the findings to other undergraduate populations. Analyses did not account for clustering or repeated measures, and there was no blinding of assessors. Despite these limitations, the study provides valuable evidence on the feasibility and effectiveness of a nurse-led, FRAMES-based brief psychosocial intervention in college settings.

## Conclusions

In this quasi-experimental, college-allocated study, a nurse-led FRAMES-based intervention was associated with greater reductions in AUDIT scores over six months versus control. By addressing the issue through brief, structured, and student-centered sessions, the intervention demonstrated significant behavioral improvements. These findings underscore the importance of integrating evidence-based, low-cost, and easily implementable interventions into college health programs, especially in areas where specialized services are hard to come by. This strategy not only tackles immediate behavioral concerns but also offers a scalable model for preventive mental healthcare. Moreover, by offering support early, it helps prevent escalation of alcohol use and, since alcohol often serves as a gateway substance, such interventions may also reduce the likelihood of future engagement with other addictive substances.

Future research should incorporate extended follow-up assessments up to one year to evaluate the persistence of intervention effects and replication with cluster-adjusted analyses. Larger and more diverse groups could be included to enhance generalizability. Comparative studies with alternative intervention models may help determine relative efficacy.

## References

[1] Mudgal SK, Patidar V, Sharma SK, Gaur R, Huda RK, Singh J, Varshney S (2025). Tobacco and alcohol use among adolescents and young adults in aspirational districts in India: NFHS-5 based secondary analysis. Pan Afr Med J.

[2] Nadkarni A, Tu A, Garg A (2022). Alcohol use among adolescents in India: a systematic review. Glob Ment Health (Camb).

[3] Debnath A, Verma A, Gupta P, Jindal S (2025). Prevalence of alcohol use disorder among medical students in New Delhi: a cross-sectional study. Indian J Community Med.

[4] Jaisoorya TS, Gowda GS, Nair BS (2018). Correlates of high-risk and low-risk alcohol use among college students in Kerala, India. J Psychoactive Drugs.

[5] Latwal GS (2020). Alcohol consumption among management students in Delhi: role of peer pressure and social norms. SSRN.

[6] Aiman S, Ahmad S (2023). Study of alcohol consumption among college students in western Punjab. Int J Acad Med Pharm.

[7] Raveendranathan D, Jaisoorya TS, Nair BS, Menon PG, Rani A, Thennarasu K, Murthy P (2020). Gender-specific correlates of alcohol use among college students in Kerala, India. Indian J Psychol Med.

[8] Carey KB, DiBello AM, Hatch MR, Weinstein AP, Neighbors C (2025). Efficacy of counter-attitudinal advocacy and personalized feedback for heavy-drinking college students. J Consult Clin Psychol.

[9] Deluca P, Coulton S, Alam MF (2020). Screening and brief interventions for adolescent alcohol use disorders presenting through emergency departments: a research programme including two RCTs. Prog Grants Appl Res.

[10] (2021). New cost-effectiveness updates from WHO-CHOICE. https://www.who.int/news-room/feature-stories/detail/new-cost-effectiveness-updates-from-who-choice.

[11] Ghosh A, Krishnan NC, Kathirvel S (2023). Digital screening and brief intervention for alcohol misuse in college students: a pilot, mixed-methods, cluster randomized controlled trial from a low-resourced setting. Asia Pac Psychiatry.

[12] Kamal K, Sunita S, Karobi D, Abhishek G (2020). Nurse-delivered screening and brief intervention among college students with hazardous alcohol use: a double-blind randomized clinical trial from India. Alcohol Alcohol.

[13] Babor TF, Higgins-Biddle JC, Saunders JB, Monteiro MG (2001). AUDIT: The Alcohol Use Disorders Identification Test: Guidelines for Use in Primary Health Care. https://www.who.int/publications/i/item/WHO-MSD-MSB-01.6a.

[14] Pal HR, Jena R, Yadav D (2004). Validation of the Alcohol Use Disorders Identification Test (AUDIT) in urban community outreach and de-addiction center samples in north India. J Stud Alcohol.

[15] Simons-Morton B, Haynie D, Liu D, Chaurasia A, Li K, Hingson R (2016). The effect of residence, school status, work status, and social influence on the prevalence of alcohol use among emerging adults. J Stud Alcohol Drugs.

[16] Benz MB, DiBello AM, Balestrieri SG (2017). Off-campus residence as a risk factor for heavy drinking among college students. Subst Use Misuse.

[17] Collins SE (2016). Associations between socioeconomic factors and alcohol outcomes. Alcohol Res.

[18] Hjarnaa L, Møller SP, Curtis AB, Becker U, Andersen O, Torvik FA, Tolstrup JS (2023). Alcohol intake and academic performance and dropout in high school: a prospective cohort study in 65,233 adolescents. J Adolesc Health.

[19] Patte KA, Qian W, Leatherdale ST (2017). Binge drinking and academic performance, engagement, aspirations, and expectations: a longitudinal analysis among secondary school students in the COMPASS study. Health Promot Chronic Dis Prev Can.

[20] Caluzzi G, Kepa K, Torney A (2025). Local environments, accessibility and affordability: a qualitative analysis of alcohol purchasing across different socio-economic areas in Victoria, Australia. Drug Alcohol Rev.

[21] Strowger M, Meisel MK, Haikalis M, Rogers ML, Barnett NP (2024). Associations between frequency of exposure to peer-generated alcohol-related posts and alcohol use within a social network of college students. Addict Behav.

[22] Kassa A, Wakgari N, Taddesse F (2016). Determinants of alcohol use and khat chewing among Hawassa University students, Ethiopia: a cross sectional study. Afr Health Sci.

[23] Mekonen T, Fekadu W, Chane T, Bitew S (2017). Problematic alcohol use among university students. Front Psychiatry.

[24] Martinez JA, Rutledge PC, Sher KJ (2007). Fake ID ownership and heavy drinking in underage college students: prospective findings. Psychol Addict Behav.

[25] Chinneikim E, Paniyadi NK, Mishra S, Panigrahi MK, Shetty AP (2024). Effectiveness of a nurse-led brief intervention on motivation toward alcohol cessation and treatment-seeking behavior. Indian J Psychiatr Nurs.

[26] Gonzalez Y, Kozachik SL, Hansen BR, Sanchez M, Finnell DS (2020). Nurse-led delivery of brief interventions for at-risk alcohol use: an integrative review. J Am Psychiatr Nurses Assoc.

[27] Platt L, Melendez-Torres GJ, O'Donnell A, Bradley J, Newbury-Birch D, Kaner E, Ashton C (2016). How effective are brief interventions in reducing alcohol consumption: do the setting, practitioner group and content matter? Findings from a systematic review and metaregression analysis. BMJ Open.

